# The Application of Rehabilitation Therapy Occupational Competency Evaluation Model in the Improvement of College Students' Innovation and Entrepreneurship

**DOI:** 10.1155/2022/7478736

**Published:** 2022-06-20

**Authors:** Zhenghan Liang

**Affiliations:** Xuchang University Innovate and Entrepreneurship College, Xuchang, Henan 461000, China

## Abstract

This paper constructs an evaluation model of occupational competency in rehabilitation therapy and applies it to the improvement of college students' innovative and entrepreneurial competencies. Based on clarifying the connotation of college students' entrepreneurial competency, this study carries out research on the evaluation system of college students' entrepreneurial competency. First, the method of qualitative research is used to conduct in-depth interviews with college students and entrepreneurial mentors to understand the views of these groups on college students' entrepreneurial competency, and the coding of the interview contents is carried out by applying the rooting theory, and finally, a theoretical model of the composition of college students' entrepreneurial competency is derived. The front-line position of talent cultivation is the construction of faculty, because the faculty with high-quality vocational ability is an indispensable prerequisite for the development of universities, and the teaching level of the faculty is the key factor for the effectiveness of teaching to appear. Based on the perspective of role theory, we analyze the problems and reasons for the lack of competency in the role-playing process; integrate the three stages of understanding the role, playing the role, and adjusting the role with the competency model; and propose strategies to improve the competency of youth social workers in the process of playing the role, to help workers accurately understand the role, correctly play the role, and reasonably adjust the role. The curriculum is designed to cultivate the competency of college student nursing positions, which is conducive to improving the professional quality of college student nursing service providers and regulating college student nursing practice so that they can actively respond to problems. This will strengthen the entire teaching faculty and enhance the professional competence of full-time teachers.

## 1. Introduction

In today's changing global economic system, whoever has the most innovative science and technology and the most sophisticated innovative talents has a head start, and countries around the world have launched innovation-driven development strategies [1]. The need of building an innovative society has given rise to the pursuit of innovative talents in the country and the important place for the cultivation of innovative talents in various universities [2]. The third is to identify the qualities that are hidden in the depths of employee traits and difficult to estimate; the last is the behavioral description that can be quantified. This need for innovative and entrepreneurial talents has further promoted the development of innovation and entrepreneurship education in colleges and universities to cultivate and improve the entrepreneurial ability of college students, and entrepreneurship education has gradually become an important aspect of the construction of double-class universities [3]. Under this environment, the methods and approaches to improve the entrepreneurial ability of college students have become the core issues of concern and consideration for scholars from all occupations in China [4]. Under the above background, the construction of the evaluation index system of the entrepreneurial ability of college students follows the principle of “promoting construction by evaluation, combining evaluation and construction, and focusing on construction,” aiming at promoting the continuous development of entrepreneurial education in colleges and universities, improving the overall entrepreneurial ability and quality of college students, relieving the employment pressure of college students, and promoting the full employment of college students [5]. Entrepreneurship education is essentially an educational activity, but because it is combined with “entrepreneurship,” entrepreneurship education becomes an educational activity with both general education and special entrepreneurship [6]. Entrepreneurship education is a present concern for students' future survival and development. Therefore, paying attention to the development of students' entrepreneurial ability means paying attention to their future development and improving their future survival and competitiveness [7]. Entrepreneurship education in colleges and universities teaches several disciplines including entrepreneurial knowledge, management knowledge, opportunity capture, marketing, professional integration, and innovation. In the process of learning and practicing, college students gradually improve their entrepreneurial ability and realize the overall development of their ability [8]. The evaluation of the entrepreneurial ability of college students is to evaluate the entrepreneurial ability of college students, to find out the weaknesses of college students in the process of entrepreneurial learning, and then pay attention to and guide them, to promote the improvement of students' ability and quality, enhancing their future survival and development ability, and realizing the overall development of students [9].

Educational evaluation is the link to make a judgment on the degree of educational activities to meet the needs of society and individuals, which should be followed up in real-time, true and accurate, comprehensive and reflective to improve, and the evaluation of college students' entrepreneurial ability can, on the one hand, capture the current college students' entrepreneurial willingness and emotional attitude, the actual development of current college entrepreneurial education, and the level of realization of college students' entrepreneurial ability; on the other hand, it can realize “promoting construction by evaluation,” discovering the deficiencies of entrepreneurship education in colleges and universities in the process of evaluation and making targeted improvements for the deficiencies and shortcomings to promote the improvement of entrepreneurship education in colleges and universities [10]. We all know what an iceberg looks like, except for the surface mountains that can be seen. Therefore, paying attention to the construction of evaluation indexes for students' entrepreneurial ability and constructing a scientific, efficient, and feasible evaluation system has become an urgent need for the development of innovation and entrepreneurship education in China's colleges and universities [11]. Teachers are the leaders of students' growth and bear important responsibilities and missions in the process of students' cultivation, while teachers' professional ability determines the overall level of the college teachers' team [12]. What professional ability contemporary college teachers need to have to meet the requirements of talented education in the fast-developing society is an urgent problem to be solved [13]. The formation of high-quality faculty is not only a path for public institutions to explore in team training but also a necessary path for some private institutions to reform their education [14]. Considering the development path of institutions themselves, private institutions need to change from ordinary colleges and universities to application-oriented universities [15].

From the perspective of the service groups faced by social workers, the problems of adolescents are more complicated and the situations are more diverse [16]. They are in the golden stage of personality growth and development; their physiology, mind, character, and will are still in the critical stage of development and have great plasticity; they need active education and guidance to promote their healthy values and outlook on life [17]. Youth social work should serve all young people to promote and counsel their development, and at the same time, it should have a strong social function [18]. It is believed that under the guidance of values, social work for adolescents is based on the unique physical and psychological characteristics of adolescent groups, and workers fully use professional social work methods and skills to help adolescents prevent and solve problems, to promote adolescents to restore their social functions and eventually achieve comprehensive development. Initially, the target of competency research was managers in enterprises, but gradually, competency research began to shift to specific industries, positions, and roles. In the study of the teachers' competency model, two research methods were adopted, namely, questionnaire survey and factor analysis, and two factors and eight levels of the teachers' competency model were constructed. Two of the factors include educational competency and collaborative competency, and the eight levels include teaching environment, teaching infrastructure, and teaching commitment.

## 2. Method

### 2.1. Design

Unlike the sample selection based on demographic variables in quantitative research, qualitative research is a rooted theory that uses theoretical sampling [19]. Theoretical sampling is the process of selecting the research subjects that can provide the maximum amount of information according to the needs of generating theories, and it is conducted simultaneously with the interview process, requiring that a large amount of unanalyzed primary data is not left in hand and that the sample selection and data analysis are conducted based on the analysis results of the initial sample and the refined theories until “theoretical saturation” is reached, i.e., the additional interview data had no effect on the addition and testing of existing categories [20]. This method can be based on specific behaviors, and the created competency model is based on reality, which is most close to the reality of the enterprise, and the application effect is good. In the sample selection of qualitative research, we generally do not pursue the quantity, but whether the collected data can adequately reflect the purpose of the study. Currently, a typical sample for a qualitative research study is usually 10-60 participants [21].

After each interview, the transcripts were converted to text word by word, making sure to restore the interviewees' answers to the greatest extent possible and to ensure the accuracy of the original material, without processing the text or adding the interviewer's subjective feelings [22]. While transcribing the transcripts, the interviewees were recorded with the interview notes or recalled the interviewees' tone of voice and expressions during the interview, and the points that were helpful for data analysis were recorded as memos [23–26]. After collecting and organizing the data, the total length of the interviews with the 20 interviewees was 660 minutes, of which the longest time was 65 minutes, the shortest time was 20 minutes, and the average length was 33 minutes, resulting in a total of 48,499 words. The interview texts were numbered one by one using the letters A-Z. In the software, we created a new project “Factors influencing the innovation and entrepreneurship ability of college students” and imported all the above 20 interview texts into this database for the next three-level coding process.

Spindle codes are more directional, selective, and conceptual than initial codes. Focus codes require judging which initial codes are most sensitive to adequately analyze the data. In this process, it is necessary to repeatedly compare and analyze the organic connection between each category, screen the main categories that best reflect the influencing factors of college students' innovation and entrepreneurship, eliminate the codes that appear less frequently and are weakly related or irrelevant to the influencing factors of college students' innovation and entrepreneurship, integrate the codes with the same or similar meanings, do unified expressions, and increase the count for the same codes [27]. Of course, this method also has shortcomings, that is, it is difficult to develop, takes a long time, and is difficult to operate. In the software, all the initial codes and the corresponding original data are repeatedly compared, the similar nodes are renamed and merged, and the codes that are irrelevant or weakly related to the influencing factors are deleted. After generalization and integration, we finally came up with 24 influencing factors of college students' innovation and entrepreneurship ability, as shown in Table 1.

The first-level index of “personal ability” mainly describes the professional ability that college students must possess in the process of entrepreneurship, mainly in terms of skills, to achieve their entrepreneurial goals. This index should reflect the characteristics of contemporary college students as well as the general characteristics of college students. According to the viewpoint of capital cultivation in the human capital theory, the first-level index, which consists of 20 three-level indicators and 6 two-level indicators, is obtained by combining expert discussion and literature review.

Innovation and entrepreneurship education is not only led by schools, promoted by teachers, and collaborated by social enterprises but also cannot be separated from students' practice. With the help of external factors such as schools, teachers, and social enterprises, students in higher education institutions should actively play their initiative, take the initiative to learn to strengthen their own innovative and entrepreneurial thinking, improve their professionalism so that they can develop their own innovative and entrepreneurial consciousness, and, at the same time, use the various resources and competition practice platforms provided by schools and society to fully demonstrate their innovative and entrepreneurial ideas and abilities on the platform [28]. At the same time, students can make use of the various resources and competition practice platforms provided by the university and society to fully demonstrate their innovative and entrepreneurial ideas and abilities on the platform and finally get the relevant innovative and entrepreneurial results.

**Figure 1:**
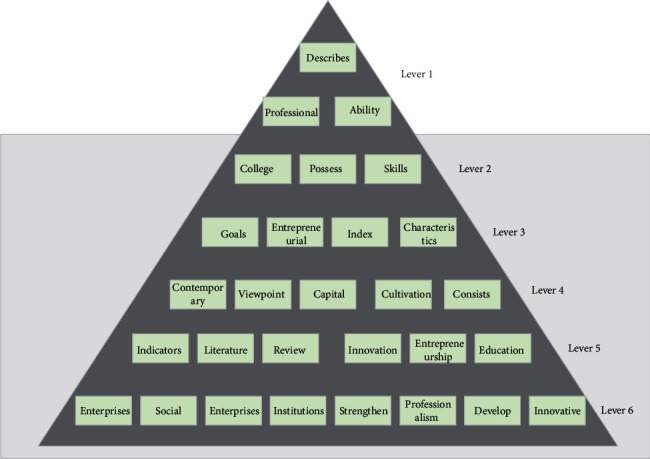
Iceberg model.

Then, the relevant personnel will select, screen out the competency items that appear frequently, and finally determine the competency model. Innovative entrepreneurship education is different from the conventional “I'll listen to you” education, and students' creativity and initiative should be fully stimulated. At the same time, the proportion of practical courses in the curriculum should be increased to strengthen students' ability to solve practical problems, as shown in Figure 1.

A role is a set of norms formed by actors in a specific context and is accomplished in long-term social interactions. A person's social role is a set of behavioral patterns and corresponding psychological states that are consistent with an individual's social status and identity and in line with social expectations. Each indicator is assigned a value of importance using the Likert 5-point scale: very important is 5, relatively important is 4, important is 3, less important is 2, and unimportant is 1 [29]. If the ability of these two parts is missing, the teaching effect will be greatly reduced, and the performance of other teaching skills will also be constrained. The expert assigns a score to each indicator according to this scale and can also modify, delete, and add to the indicators in the preset modification and additional columns.

Competency models are characterized by, first, core characteristics that distinguish top performers from average performers; second, they can effectively establish talent standardization; third, they can identify the difficult-to-measure qualities hidden in the depths of employee traits; and finally, they can quantify behavioral descriptions. We all know what an iceberg looks like. In addition to the surface mountain that can be seen, there is also the huge part hidden under the water surface that is difficult to measure. The underwater part of the ice is difficult to measure and contains many aspects such as traits, motivation, and values. This part of the underwater competency characteristics is like the lower part of the iceberg that is not easily changed by the external environment, so it is more difficult to assess and improve, but it is the part of the talent. It is also the core content of the “iceberg model” theory research.

This method can be based on specific behaviors, and the competency model created is based on reality, closest to corporate reality, and can be applied with good results [30]. Of course, this method also has shortcomings, namely, it is difficult to develop, takes a long time, and is somewhat difficult to operate. It requires the ability to interview behavioral events and to determine the competency model by studying the differences between high-performing employees and average performers.

The next method is the derivation method, which is accurately described as a logical derivation process. Among them, the working years between 4 and 8 years accounted for 76.4% of the total number of samples. The competencies are derived by clarifying the organizational vision and strategy and understanding the job roles and responsibilities. The advantage of this research method is that the competency model is closely related to the company's strategy and core values, and the logical line of analysis will be very clear. The disadvantage is that it is too abstract, without concrete behaviors to support the basis and easily detached from reality.

Finally, there is the revision method, which is an easy and simple way of research. Usually, it is a preliminary understanding of the enterprise organization by professionals, combined with the generic competency model, a considerable number of competency items are proposed, and then, the selection is made by the relevant personnel to filter out the high frequency of competency items and finally determine the competency model. The advantage of this approach is that it saves time and effort and is efficient and convenient, but the disadvantage is also obvious that there are too many generic components and a lack of relevance.

Based on the philosophy of human resources education and training at CM College and the professional competency of teachers at CM College, I used the method of deduction, induction, and revision to construct a “dual-teacher” teacher competency model in line with contemporary education, so I changed the dimensions and added the comprehensive practice dimension of practice and teacher-student collaboration [31]. Currently, newly recruited student counselors generally work as students for several years and then choose to continue their studies to transfer to teachers or administrative positions. The four dimensions of the teacher competency model are integrated into the professional competency of teachers, and the research on the development of teachers' professional competency to “dual-teacher” teachers is carried out from the aspects of personal characteristics, teaching attitude, teaching skills, and comprehensive practice. In particular, the fourth dimension of teachers' professional competency, “comprehensive practice ability,” is highlighted in the context of the current social demand for talents, and the specific competency elements under each dimension are shown in Table 2.

From the above analysis, we can conclude that the competencies of “dual-teacher” teachers can be composed of four dimensions, namely, personal characteristics, teaching attitude, teaching skills, and comprehensive practice, which include a total of 40 competencies. In addition to the three existing dimensions, another important dimension has been added [32]. Five competencies were added to the personal attributes, including sensitivity, extroversion, fairness, emotionality, and creativity, among which creative personal attributes play an important role in today's education and teaching. Three competencies were added to the teaching attitude, including moral education, love, and being a teacher, in which moral education is fundamental to students' development [33]. The two competency elements of lecture expression ability and teaching management ability are added to the teaching skills. In this big environment, the methods and ways of improving the entrepreneurial ability of college students have also become the core issues that scholars from all walks of life in our country pay attention to and think about. If these two parts of the competency are missing, the teaching effect will be greatly reduced, and the performance of other teaching skills will be hampered. The other dimension is comprehensive practice, such as cross-border ability, integration ability, and ability to transform results, which is an important impetus to the development of professional competence of “dual-teacher” teachers.

### 2.2. Experiment

The subjects of this study are college students, and the main reason for choosing this organization is based on the following considerations: first, the organization is representative. It is the second batch of national social work service demonstration units and the national training base for social work professionals in youth affairs and has a group of experienced youth social workers who are mature in the field of youth social work. Since the behavioral event interview method requires good work performance of the interviewees, the criteria for selecting the subjects in this study were (1) at least 2 years of practice in the agency and 2 years or more of social work experience, (2) graduation in social work, and (3) having social worker qualification. Second is the convenience of data collection [34]. Our university and the organization are committed to promoting the localization of social work and building a long-term cultivation mechanism with the industry promotion mode of “university + organization,” which links more advantageous resources for students and provides convenience for data collection. Due to the author's interest, ability, and familiarity with the region, social work agencies were chosen to conduct the study of competency issues [35].

Second, the interviewees were identified and communicated with in advance; their willingness to be interviewed was sought; and the time, place, and format of the interview were agreed upon. Again, the purpose of the interview and its content were informed before the interview, the interview was recorded with the consent of the interviewee and kept confidential, and the length of the interview ranged from 50 to 90 minutes. Finally, after the interviews were completed, the author promptly organized the content of the interviews and asked follow-up questions about any unclear or undetailed content [36]. After completing the interview, the author reflected on the content and communicated with the supervisor to facilitate timely correction and adjustment of any deficiencies and to ensure the quality of the interview. In addition, the process of organizing the interview data is also the process of analyzing and coding, which helps to select the next theoretical sampling interviewees.

**Figure 2:**
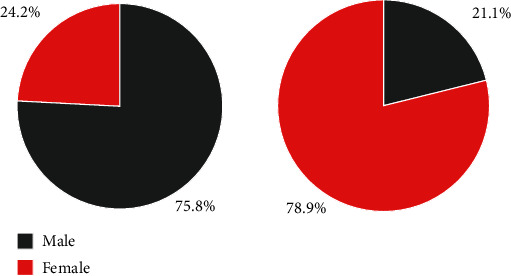
Distribution of the gender structure of the sample.

In this study, the interview data were coded and organized mainly using the three-level coding of procedural rooting theory and NVIVO12 analysis software. Based on the Zagan theory paradigm to analyze the obtained raw data, the raw interview data of the interviewees were initially conceptualized and labeled as a1-aX, the categorized concepts were coded as A1-AX, and the main categories were coded as AA1-AAX to facilitate coding statistics and organization. NVIVO12 (Chinese version) was borrowed for this process as supplementary analysis software, which cannot replace the human brain to think about coding, but the coder can use search queries, a summary of relevant node information to systematically think and organize the same coding dimension, and can use mind maps to construct conceptual models, and can use annotations, memos, etc. to record detailed ideas and reflect deeply on the connections between nodes, etc. These features greatly improve the efficiency and effectiveness of analysis [37]. All these functions greatly improve the efficiency and accuracy of analysis, as shown in Figure 2.

In this sample survey, there were 87 male counselors, accounting for 42.6% of the total sample size, and 117 female counselors, accounting for 57.4% of the total sample size. The distribution of the sample is reasonable and in line with the current gender structure of full-time student counselors in private colleges and universities.

Among them, 33 have worked for less than 4 years, accounting for 16.2% of the total sample; 119 have worked for 4-7 years, accounting for 58.3% of the total sample; 37 have worked for 8-10 years, accounting for 18.1% of the total sample; 15 have worked for more than 10 years, accounting for 7.4% of the total sample. Since the questionnaires were selected for full-time student counselors and some deputy secretaries in charge of student work, the distribution of working years is consistent with the current distribution of working time of counselors in colleges and universities, in which those with working years between 4- and 8-years account for 76.4% of the total number of the sample [38]. Entrepreneurship education has become an educational activity with both general education and entrepreneurial specificity. Entrepreneurship education is the current concern for students' future survival and development. The basic years of experience can better reflect the actual situation of student counselors in real student work, and the sample is more malleable and time-sensitive.

Research on competency has been fruitful both in the scope of theoretical research and in the scope of applied research. Experts and scholars have explored the connotation and division of competencies mainly in terms of knowledge, abilities, and personal traits, and the research on competency models is mainly divided into the iceberg model and the onion model, based on which competency models are classified and studied in different industries. Researchers who have explored the competency model include psychologists, management scientists, and human resource experts who have conducted in-depth research on the competency model and applied it to various fields.

Among them, the talent competency model of the tourism industry is also included, but specifically, there are some weak aspects in the research about the tour guide competency model, such as most scholars just keep the tour guide competency model in the theoretical research aspect, without applying it to practice or specific teaching, so this study takes the competency model as the basis and constructs the tour guide competency model through various methods, hoping that it can provide a reference for the future tourism industry [39]. This study, therefore, constructs a tour guide competency model based on the competency model through various methods, hoping that it can provide a reference for the training and recruitment of tour guides in the future tourism industry.

Expertise refers to the knowledge base necessary to practice the social work profession, which provides a systematic knowledge structure for the development of services. Youth social workers should master the specific role knowledge from the beginning of playing the role to accurately understand the behavioral norms of the role. The knowledge that a qualified youth social worker should have can be broadly classified into three categories: basic theoretical knowledge of youth social work, social welfare, policy, multidisciplinary knowledge, etc. During the interviews, all eight interviewees mentioned the importance of this category.

Theoretical knowledge serves as a guide for service development, and the degree of workers' comprehension and ability to apply it directly affects the choice of service actions. In descending order of the number of reference points, the theories frequently used by the respondents were dominance perspective, psychosocial theory, humanistic theory, and cognitive behavioral therapy theory, as shown in Table 3.

The importance of adding an entry on professional ethics was assigned a score of 5. After discussion, the group considered the special nature of geriatric nursing work and agreed to add an entry on professional ethics because they believed that geriatric nursing should have good professional ethics. B8 analytical assessment ability belongs to the thinking aspect and belongs to inappropriate. The group discussed and decided to delete the analysis and assessment ability [40]. In the process of learning and practice, college students gradually improve their entrepreneurial ability and realize the all-round development of their own ability. The Academic Affairs Office of the university coordinates the opening of elective courses related to innovation and entrepreneurship, while each second-level faculty offers innovation and entrepreneurship education with its professional characteristics, to improve students' comprehensive professional ability.

Innovation and entrepreneurship can never be accomplished by one person independently. From the establishment process of Alibaba, we can see that the strength of a team is crucial in the early stage of entrepreneurship, and as a student, it is even more important to form a good team to participate in the innovation and entrepreneurship competition. However, it is easy to see from the performance of student's daily life in recent years that college students in the new era care more about their feelings and tend to selectively ignore the feelings of others, so improving students' team organization ability and team spirit is a key factor to ensure that students can go far in the innovation and entrepreneurship competition.

## 3. Result

### 3.1. Models

**Figure 3:**
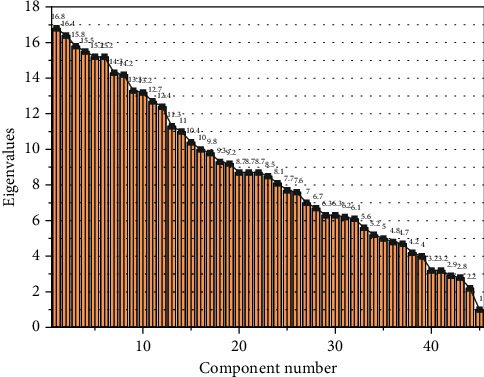
Fragmentation diagram of the factor.

In this study, the questionnaire of scale type question items is analyzed and studied by principal component analysis, and the results of the analysis are shown in Figure 3. From the Figure 3 gravel plot, the curve appears more obvious changes at the ninth factor, that is, the curve goes flatter and the curve has a more obvious inflection point. Therefore, this principal component analysis of the questionnaire can temporarily retain the first 9 factors according to the performance of the gravel plot. According to the results of the total variance of the variables explained by the factors, the cumulative variance explained by the first 9 factors reached 68.42%, indicating that the first 9 public factors could barely reflect the information of the original variables. From Figure 3, we can see that the cumulative variance of the factors did not change after rotation, indicating that the commonness of the variables did not change after rotation, only the variance explained by each factor of the original variables was redistributed, and the variance contribution of each factor has changed, thus making the factors more explanatory.

The orthogonal rotation of the factor-loading matrix by the variance-maximization method makes the factors have named interpretations, and thus, the named interpretation of the factors facilitates the interpretation and evaluation of the factor analysis results. After rotating the factor-loading matrix, the rotated factor-loading matrix is derived. According to the factor-loading coefficients of the rotated factor-loading matrix, the variable items in the rotated component matrix were sorted, and a rotated factor-loading matrix loading of 0.35 or greater was generally considered to be meaningful. The evaluation of entrepreneurial ability of college students is to evaluate the entrepreneurial ability of college students, to find the weaknesses of college students in the process of entrepreneurial learning. The rotated factor-loading matrix was compiled and initially classified according to the rotated factor loadings matrix.

In this study, the number of public factors was selected and optimized by combining the logical correlation between the public factors derived from factor analysis and the public factors of each index element were named. In this study, factor 1, factor 2, factor 3, factor 4, and factor 6 were selected as public factors.

Since both factor 5 and factor 1 can explain attitude quality in explaining counselor competency, factor 5 and factor 1 are combined into attitude quality. In other words, attitude quality can be used to actively participate in students' classroom activities; integrate into students' communities; deal with students' affairs fairly, justly, and objectively; learn to think differently about the particularity of minority students; and be willing to cooperate with others to complete the work of students of all nationalities. Take minority students as the center, do a good job in service management, have confidence and expectations for each minority student, and listen to the opinions of minority students patiently, carefully, and attentively. The explanatory variables include seriousness and nonpartisanship in implementing school rules and regulations, setting an example to educate and influence students, effectively controlling emotions and maintaining good working conditions.

Since factor 7, factor 8, and factor 3 can all explain individual traits in explaining counselor competency, factor 7, factor 8, and factor 3 combined into individual traits. That is, individual traits can be explained by variables such as actively conducting research on students' ideological and political education, desiring to excel or exceed the standard of excellence in work performance, making work plans to ensure smooth work, actively desiring to learn new knowledge and master new skills, conscientiously fulfilling counselor obligations and completing various tasks, facing the work of students in ethnic colleges and universities with untiring and dedicated work, and loving counselor work without complaining or grumbling.

### 3.2. Applications

Execution refers to the ability of workers to transform existing knowledge and skills into specific behaviors through practice so that universal knowledge and skills can be presented in specific scenarios and cases. This is a process of applying theory to practice, which requires high comprehensive quality of workers and requires them to make corresponding behavioral strategies according to specific scenarios, proficiency of knowledge and skills, and their behavioral habits. The professional ability of teachers themselves determines the overall level of the teaching staff in colleges and universities. This is also the stage of balancing the ideal role with the real role. This comprehensive action power of youth social workers is mainly manifested in the worker's ability to adapt to the environment, situational observation, creativity, and writing ability. In situational observation, observation is a purposeful and persistent perceptual process, which plays an important role in the process of the worker's particular situational practice. More observant people are good at finding connections between things around them and perceiving the less obvious features of things keenly.

The individual level contains 10 dimensions of interest, personality, career planning, survival needs, ideal belief, achievement motivation, entrepreneurial enthusiasm, human network, entrepreneurial learning, and practice. Interest can stimulate the initiative of innovation and entrepreneurship of college students, and when they are interested in innovation and entrepreneurship, college students will take the initiative to improve their innovation and entrepreneurship ability. Students who have a good personality or a personality consistent with innovation and entrepreneurship are more inclined to choose innovation and entrepreneurship and then develop innovation and entrepreneurship ability. By incorporating innovation and entrepreneurship into career planning and clarifying the development goals, college students can target to develop the corresponding abilities, as shown in Table 4.

People's survival needs, ideal beliefs, and achievement motivation will drive them to work hard subjectively and improve their ability as soon as possible to achieve success in innovation and entrepreneurship. College students with high entrepreneurial enthusiasm will keep working hard and persisting for the cause they like, which has a positive effect on their innovative and entrepreneurial ability. Innovative entrepreneurship requires a lot of human resources, and to have enough strong human resources, requires their innovative entrepreneurial ability. It is an urgent problem to be solved which professional ability modern college teachers need to have to meet the requirements of the rapidly developing society for talent education. And entrepreneurship learning and practice are to acquire entrepreneurship-related knowledge, accumulate innovation and entrepreneurship experience, and enhance innovation and entrepreneurship ability. Among them, entrepreneurial learning includes book learning, professional learning, entrepreneurial knowledge learning, social learning, and prior experiential learning. Practice includes student work experience, part-time experience, social practice, and entrepreneurial practice. Among all types of practice, innovation and entrepreneurial practices are the most obvious positive influence on college students' innovation and entrepreneurial ability and the most effective way.

**Figure 4:**
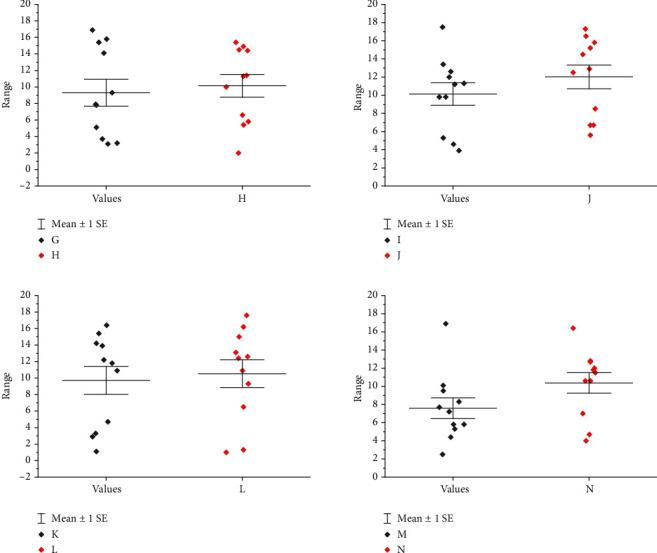
The situation related to the innovation and entrepreneurship ability of college students.

As shown in Figure 4, the university level includes 6 dimensions of mentorship, innovation and entrepreneurship atmosphere, innovation and entrepreneurship curriculum, innovation and entrepreneurship activities, innovation and entrepreneurship competition, and innovation and entrepreneurship club, which can be collectively referred to as innovation and entrepreneurship education. The most important one is mentorship, which has an important guiding role in the formation and development of college students' innovation and entrepreneurship ability, including on-campus mentors such as tutors, teachers of professional courses and counselors, and off-campus mentors with entrepreneurial experience such as entrepreneurs, professional managers, and entrepreneurs. On-campus mentors have more influence on innovators and those who transform scientific and technological achievements, and off-campus mentors have more influence on entrepreneurs. It is believed that youth social work is guided by values, and workers make full use of social work professional methods and skills to help youth prevent and solve problems according to the unique physical and psychological characteristics of youth groups, to promote youth to restore their own social functions. A good innovation and entrepreneurship atmosphere helps to enhance the innovation and entrepreneurship ability of college students, which specifically includes the importance of innovation and entrepreneurship, system construction, atmosphere creation, policy support, and other aspects of the school. Innovation and entrepreneurship courses are an indispensable part of innovation and entrepreneurship education at the college level, while innovation and entrepreneurship activities, innovation and entrepreneurship competitions, and innovation and entrepreneurship clubs all provide platforms and services for students' innovation and entrepreneurship practice.

Meanwhile, innovation and entrepreneurship competitions have positive and significant correlations with all other secondary and tertiary dimensions of innovation and entrepreneurship ability, except for the absence of correlation with interpersonal ability. This factor verifies the school-level factors influencing innovation and entrepreneurship competitions in the previous model of factors influencing college students' innovation and entrepreneurship competencies. While transcribing the transcript, combine the records of the interview, or recall the tone, demeanor, and facial expressions of the interviewee during the interview. Both the qualitative and quantitative studies affirm the influence of innovation and entrepreneurship competition on college students' innovation and entrepreneurship ability, and the results of both validate each other.

There is a significant positive relationship between professional achievement ranking and other secondary and tertiary dimensions of innovation and entrepreneurship ability. Students' professional achievement ranking reflects professional learning, which verifies the factors influencing entrepreneurial learning at the individual level in the previous model of factors influencing college students' innovation and entrepreneurship ability. Several respondents in the qualitative study mentioned that the improvement of innovation and entrepreneurship ability influenced professional learning, and they give priority to their majors in the process of conducting innovative practice. The positive correlation between the ranking of major performance and the innovation and entrepreneurship ability of college students in the quantitative study precisely verifies the conclusion of the qualitative study.

## 4. Discussion

At the social level, there is a positive and significant correlation between the place of origin and all the innovation and entrepreneurship competencies except the competencies under the three tertiary dimensions; at the school level, there is a significant positive correlation between the innovation and entrepreneurship curriculum and innovation and entrepreneurship activities and all the competencies under the structure framework of innovation and entrepreneurship competencies of college students, and innovation and entrepreneurship competitions and innovation and entrepreneurship clubs are significantly and positively correlated with all the innovation and entrepreneurship competencies except the competencies under one tertiary dimension. At the family level, there are significant positive correlations between parents' highest education, family innovation and entrepreneurship experience, and family support and all competencies under the structural framework of college students' innovation and entrepreneurship competencies; and there are significant positive correlations between only children and all competencies under the three levels of competencies; at the individual level, there are significant positive correlations between student cadre experience, entrepreneurship experience, and all competencies under the structural framework of college students' innovation and entrepreneurship competencies. The shortest time was 20 minutes, the average time was 33 minutes, and a total of 48,499 words of interview text were formed. There is a significant positive correlation between all competencies in the structural framework, and there is a significant positive correlation between professional performance ranking and all innovative entrepreneurial competencies except those in the three three-level dimensions; while there is no correlation between internship and part-time job experience and family economic situation and innovative entrepreneurial competencies of college students, that is, internship and part-time job experience and family economic situation do not influence innovative entrepreneurial competencies of college students.

## 5. Conclusion

In this study, we constructed and measured a competency model for the rehabilitation therapy profession to derive credible competency elements and models for college students. The competency model for youth social workers was constructed using a rooted theory research approach. A total of 6 main categories and 23 domains were included, including professional knowledge including theoretical knowledge of youth social work, multidisciplinary knowledge, social welfare, and policy; professional skills including language and communication skills, home-school-social organization and coordination skills, resource integration skills, case management, and crisis management skills; personal traits including affinity, positive optimism, responsibility, and risk-taking spirit; and professional values including empathy, individualization, and individualization. Professional values include empathy, individualization, and potential and development; executive skills include innovation, environmental adaptation, situational observation, and writing skills; and professional reflexivity includes critical thinking, subjectivity, social work imagination, and professional empowerment. The model reaches the state of theoretical saturation when no new concepts and categories are derived through the theoretical saturation test. Combining expert discussions and literature review, a first-level indicator consisting of 20 third-level indicators and 6 second-level indicators was obtained. At the same time, the development of college students' entrepreneurial ability can promote social employment, provide more impetus for social and economic development, and stimulate social creativity kinetic energy. This study is based on the evaluation of college students' entrepreneurial ability, to achieve the improvement of college students' entrepreneurial ability through evaluation, prompting them to develop their comprehensive ability, adapt to the ever-changing information society, and enhance their competitiveness. Educational evaluation is the baton of educational reform, guiding the direction and pace of educational reform. The research on the evaluation of college students' entrepreneurial ability can realize the evaluation to promote education, promote the development of college entrepreneurial education, realize the perfection and systematization of college education, and then enhance the educational competitiveness of colleges and universities.

## Figures and Tables

**Table 1:** Components of the entrepreneurial ability of college students.

Serial number	General	Integral	Serial number	General	Integral
1	4	13	13	5	11
2	2	19	14	5	19
3	2	13	15	5	12
4	3	20	16	3	20
5	3	16	17	3	14
6	4	17	18	3	13
7	5	17	19	2	20
8	3	15	20	4	11
9	5	11	21	2	17
10	3	17	22	5	15
11	5	17	23	2	14
12	5	18	24	4	14

**Table 2:** Occupational quality competencies.

Dimension	Competence item enterprising spirit	Specific behavior
1	Loyalty	Not satisfied with the status quo, set higher work goals, and persistently pursue a vigorous and upward mental state toward new goals.
2	Antistress	Dedicated, sincere and selfless, refers to the behavioral orientation and psychological belonging to the enterprise, and the degree of dedication to the work.
3	Appeal	The ability to resist pressure, can adapt well, adjust, be active, and have an unyielding heart under various blows.

**Table 3:** Results of professional literacy expert consultation.

Secondary indicators	Average value	Standard deviation	CV (%)	Full score ratio (%)
B1	7.11	3.51	43.9	23.1
B2	6.05	1.95	45.1	69.6
B3	9.72	9.44	64.5	54.5
B4	8.72	9.06	62.2	58.1
B5	7.46	8.82	45.9	54
B6	3.89	5.07	32.7	42.2
B7	2.17	3.99	10.1	40.9
B8	5.14	9.53	48.6	77.6
B9	7.87	6.48	57	49.3

**Table 4:** Summary of interview reliability feedback.

Number	Respondents	Interview authenticity	Content compliance
1	A	10	9.4
2	B	10	10
3	C	10	9.8
4	D	10	9.5
5	E	10	10
6	F	10	9.4
7	G	10	10
8	H	10	9.7
9	I	10	9.4
10	J	10	10

## Data Availability

The data used to support the findings of this study are available from the corresponding author upon request.
